# Tubarial salivary glands show a low relative contribution to functional salivary gland tissue mass

**DOI:** 10.1007/s12149-024-01965-x

**Published:** 2024-07-26

**Authors:** Sui wai Ling, Astrid van der Veldt, Marcel Segbers, Henk Luiting, Tessa Brabander, Frederik Verburg

**Affiliations:** 1 Department of Radiology and Nuclear Medicine, Erasmus MC, Dr. Molewaterplein 40, 3015 GD Rotterdam, the Netherlands; 2 Department of Medical Oncology, Erasmus MC, Rotterdam, the Netherlands; 3 Department of Urology, Erasmus MC, Rotterdam, the Netherlands

**Keywords:** Tubarial salivary glands, PSMA, Radionuclide therapy, Actinium, Lutetium

## Abstract

**Background:**

In 2021, the tubarial salivary glands (TSGs) were newly identified on prostate-specific membrane antigen (PSMA) positron emission tomography/computed tomography (PET/CT) as macroscopic glands in the nasopharyngeal wall. However, the relative contribution of the TSGs to the total salivary gland function, and consequently on the development of xerostomia after external beam radiotherapy (EBRT) or PSMA-targeted radionuclide therapy (RNT) is not known. Therefore, we aimed to determine the presence of the TSGs and to quantify uptake in the TSGs on PSMA PET.

**Methods:**

Qualitative and quantitative analyses were performed on ^68^Ga-PSMA-11 PET/CT scans of 100 patients with prostate cancer. The mean and maximum standardized uptake value (SUVmean and SUVmax) in the TSGs were measured and compared to the parotid, submandibular and sublingual salivary glands (PSGs, SMSGs and SLSGs, respectively). Furthermore, proportional function of the TSGs was compared to the PSGs, SMSGs and SLSGs based on the total organ PSMA (TO-PSMA).

**Results:**

The TSGs were visible on 95% of the ^68^Ga-PSMA-11 PET/CT scans. The normalized median SUVmean and SUVmax was significantly higher for the PSGs (*p* < 0.001) and SMSGs (*p* < 0.001) compared to the TSGs, but not for the SLSGs (*p* = 0.242 and *p* = 0.300, respectively). The normalized median TO-PSMA was significantly higher for the PSGs (*p* < 0.001) and SMSGs (*p* < 0.001), and significant lower for the SLSGs (*p* < 0.001) compared the TSGs.

**Conclusions:**

The SUVmean, SUVmax and TO-PSMA of the TSGs were most comparable to the SLSGs. However, the measured PSMA uptake may be disproportional towards the saliva production. Therefore, future studies should focus on the relation between PSMA uptake and salivary function before and after PSMA therapy.

## Introduction

In recent years, prostate-specific membrane antigen (PSMA)-targeted radionuclide therapy (RNT) has shown promising results in patients with metastatic castration-resistant prostate cancer (mCRPC) [1]. PSMA is a type II transmembrane glycoprotein (also named glutamate carboxypeptidase II or folate hydrolase I) which mainly shows expression on both benign prostate epithelium and prostate cancer (PCa) cells, but expression is also observed in healthy tissues such as the kidneys, small intestine and the salivary glands [2–7]. However, PSMA expression on PCa cells is up to a thousand-fold higher than on healthy tissues, especially in more advanced PCa [8, 9].

Although patients with mCRPC could benefit from PSMA-targeted RNT, patients also have adverse events with xerostomia being the most frequently reported adverse event [1]. Xerostomia can be explained by the physiological high PSMA expression in the salivary glands resulting in a significant radiation dose after RNT [6, 7]. Previously, dosimetry of Lutetium-177-PSMA (^177^Lu-PSMA) identified the salivary glands as dose-limiting organs for RNT with ^177^Lu-PSMA [10–12]. Furthermore, the clinical relevance also extends to patients with head-and-neck cancer or brain metastasis as high-dose external beam radiotherapy (EBRT) could lead to xerostomia [13]. Saliva protects the oral mucosa by inhibiting bacterial overgrowth and supports remineralization of dental hard tissue [14]. In addition, saliva is essential for taste perception and speech. As a result, dysfunction of the salivary glands may lead to patient discomfort (such as a subjective feeling of a dry mouth, dysgeusia or problems with speech or chewing) and negatively impact the quality of life [14].

Besides the parotid salivary glands (PSGs), the submandibular salivary glands (SMSGs) and the sublingual salivary glands (SLSGs), the tubarial salivary glands (TSGs) were identified in 2021 as a “new” organ-at-risk for radiation burden. The TSGs are located at the posterior in the nasopharynx [13] and also have PSMA expression [7, 15]. However, the impact of these TSGs on the development of xerostomia after PSMA-targeted RNT is not known. Therefore, the aim of this study was to determine the presence of the TSG on PSMA positron emission tomography/computed tomography (PET/CT) and to quantitatively compare the PSMA uptake between the TSGs and the PSGs, SMSGs and SLSGs.

## Methods

### Patient population

For this analysis, one hundred ^68^Ga-PSMA-11 PET/CT scans were randomly selected from the database of the imPRINT trial (NL7165 (NTR7389)). The imPRINT trial is a prospective study which included patients with PCa who underwent ^68^Ga-PSMA PET/CT for initial staging or after disease progression [16]. All patients gave written informed consent. The study was approved by the ethical committee in the Netherlands (MEC-2016–519).

### Imaging acquisition

PSMA-11 was labeled with Gallium-68, and ^68^ Ga-PSMA-11 PET/CT images were acquired for standard patient care. The injected activity of ^68^ Ga-PSMA-11 was 1.5 MBq (0.04 mCi)/kg and 20 mg of furosemide was injected prior to the administration of the tracer. Scans were performed on a Biograph PET/CT (Siemens Healthineers, Erlangen, Germany) approximately 1 h after the injection of ^68^Ga-PSMA-11. Patients were instructed to void immediately before the start of acquisition. The ^68^Ga-PSMA-11 PET images were performed from skull base to the proximal femora. The PET acquisition was performed at 3 min per bed position for patients ≤ 70 kg and 4 min for patients > 70 kg. The PET images were reconstructed using an iterative reconstruction method with 3 iterations, 24 subsets, 200 × 200 matrix, Gaussian filter and FWHM of 6.0 with a CT-based attenuation correction (dose modulated low-dose CT, 30 ref mAs, 3.0 mm reconstruction) according to the EARL guidelines [17].

### Qualitative and quantitative analyses

First, qualitative assessment was performed to determine whether the TSGs was visible and showed uptake of ^68^Ga-PSMA-11. In addition, the uptake of ^68^Ga-PSMA-11 in the TSGs was compared qualitatively with the uptake in the PSGs, SMSGs and SLSGs and categorized into three categories: “less uptake”, “comparable uptake”, and “higher uptake”.

For the quantitative assessment, the mean and maximum standardized uptake value (SUVmean and SUVmax, respectively) of the PSGs, SMSGs, SLSGs and TSGs were measured and compared. The largest cranio-caudal length of the TSGs was measured mainly on the sagittal slice; however, if the diameter was not clear in this projection, the coronal slice was used for measurements. All measurements were performed on the ^68^ Ga-PSMA-11 PET images. The normalized SUVmean and SUVmax were defined as SUVmean and SUVmax of the salivary glands divided by the SUVmean and SUVmax of the background value, i.e., thoracic aorta blood pool, respectively. Furthermore, contribution per salivary glands was measured by contouring each salivary gland. PSMA-derived organ volume (PSMA-OV) was calculated by drawing volume of interests around each salivary gland separately using a 40% cutoff of the SUVmax which was based on the literature [18]. Subsequently, the total organ PSMA (TO-PSMA) was calculated by multiplying the normalized SUVmean with the PSMA-OV. The functional contributions of the TSGs were calculated by dividing the TO-PSMA of the TSGs to the total TO-PSMA (PSGs, SMSGs, SLSGs and TSGs) and then multiply by 100. The qualitative and quantitative assessments were performed by SL. All measurements and analysis were performed using OsiriX MD (version 8.0.2) and Mirada Simplicity90® (version 2.4).

#### Statistical analysis

All continuous data were reported as median and the 25th and 75th interquartile ranges. For the comparison of the median SUVmean, SUVmax and TO-PSMA between the TSG and the PSG, SMSG and SLSG, the Wilcoxon signed ranks test was used. Statistical analyses were performed using SPSS (version 28.0.1.0, IBM Corp., Armonk, NY, USA).

## Results

The median age of the selected population (*n* = 100) was 71 years (IQR 63–74) and the median injected activity of ^68^ Ga-PSMA-11 was 123 MBq (IQR 108–136), equivalent to 3.3 mCi (IQR 2.9–3.7).

**Fig. 1:**
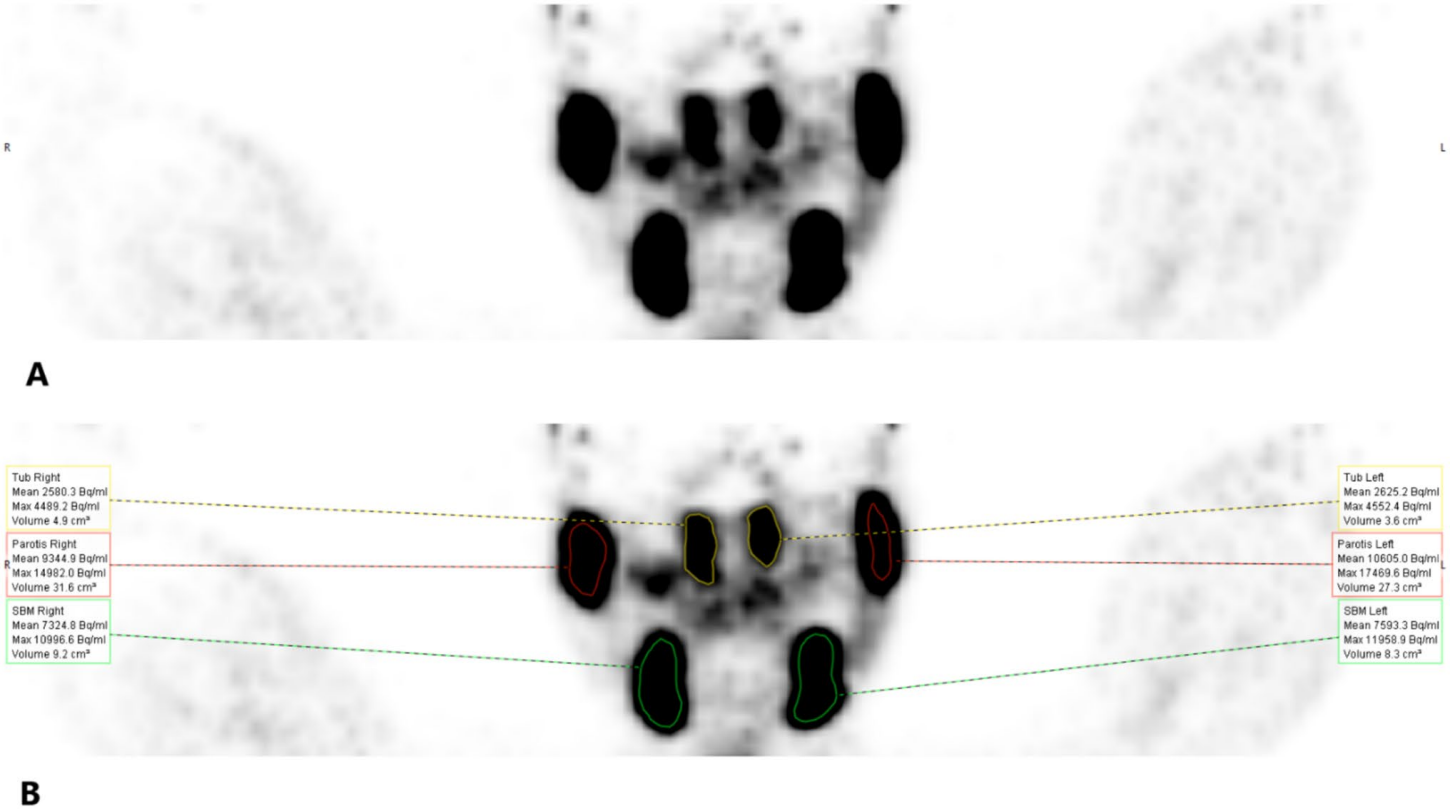
Example of the segmentation of the parotid, submandibular and tubarial salivary glands. **A** Pre-segmentation. **B** Post-segmentation

The TSGs were visible on 95% of the ^68^Ga-PSMA-11 PET/CT scans (*n* = 100). The median of cranio-caudal length of the TSGs (*n* = 95) was 37.95 (IQR 28.89–44.78) millimeters (mm). In 71 out of 95 (74.7%) of the TSG, ^68^Ga-PSMA-11 uptake was comparable with the major salivary glands, whereas lower uptake was observed in 24 out of 95 (25.3%) of the TSGs (Example in Fig. 1).

The normalized median SUVmean was 6.1 (IQR 5.2–8.3), 6.6 (IQR 5.3–8.2), 2.2 (IQR 1.4–3.0) and 2.0 (IQR 1.6–2.4) for the PSGs, SMSGs, SLSGs and TSGs, respectively. The normalized median SUVmax was 12.0 (IQR 10.2–15.0), 12.4 (IQR 10.3–15.5), 4.2 (IQR 2.9–6.0) and 4.2 (IQR 3.2–4.8) for the PSGs, SMSGs, SLSGs and TSGs, respectively (see Table 1). The normalized median SUVmean and SUVmax were significantly higher for the PSGs (*p* < 0.001) and SMSGs (*p* < 0.001) compared the TSGs. There was no significant difference between the normalized median SUVmean and median SUVmax of the TSGs and SLSGs (*p* = 0.242 and *p* = 0.300*,* respectively).

**Table 1:** Normalized SUVmean, SUVmax and TO-PSMA values

	SUVmean (median (IQR))	*P* value	SUVmax (median (IQR))	*P* value	TO-PSMA (median (IQR))	*P* value
Parotid SGs	6.1 (5.2–8.3)	< 0.001	12 (10.2–15)	< 0.001	190.9 (138–240.1)	< 0.001
Submandibular SGs	6.6 (5.3–8.2)	< 0.001	12.4 (10.3–15.5)	< 0.001	70.1 (50.2–83.4)	< 0.001
Sublingualis SGs	2.2 (1.4–3)	0.242	4.2 (2.9–6)	0.300	7.5 (4.9–10.3)	< 0.001
Tubarial SGs	2 (1.6–2.4)	–	4.2 (3.2–4.8)	–	10.6 (8.6–14.5)	–

*IQR* interquartile range, *PSMA* prostate-specific membrane antigen, *SGs* salivary glands, *SUVmax* maximum standardized uptake value, *SUVmean* mean standardized uptake Value, *SD* standard deviation, *TO-PSMA* total organ PSMA

The normalized median TO-PSMA was 190.9 (IQR 138.0–240.1), 70.1 (IQR 50.2–83.4), 7.5 (IQR 4.9–10.3) and 10.6 (IQR 8.6–14.5) for the PSGs, SMSGs, SLSGs and TSGs, respectively (see Table 1). The normalized median TO-PSMA was significantly higher for the PSGs (*p* < 0.001) and SMSG (*p* < 0.001), and significant lower for the SLSGs (*p* < 0.001) compared the TSGs. The median functional contribution of the TSGs was 4.3% (IQR 3.3–5.4) of the total salivary glands function.

## Discussion

Although the TSGs have extensively been described by Valstar et al. [13], no quantitative assessment of these glands was performed. To the best of our knowledge, we were the first to assess the proportional function of the different salivary glands by measuring the TO-PSMA to estimate the salivary gland function more precisely. Sakthivel et al. [15] were the first to illustrate the physiological uptake of ^68^ Ga-PSMA in the PSGs, SMSGs, SLSGs and the TSGs by reporting the median normalized SUVmax values, which were comparable to our findings (see Table 2). The PSGs and SMSGs showed the highest SUVmax, while a lower SUVmax was observed in the SLSGs and TSGs. Therefore, Sakthivel et al. [15] suggested that the TSGs could only be considered as aggregates or minor salivary glands due to gland volume and functional capacity of the gland which is associated with the PSMA uptake. This finding was further supported by the data that Valstar et al. [13] published in which they showed that the TSGs were similar to the SLSGs due to the primarily mucous aspect without amylase expression and very low numbers of serous acini. Based on our TO-PSMA analysis, the salivary gland function of the TSGs would also be most comparable to the SLSGs.

**Table 2:** Comparison of normalized SUVmax values

	Our study		Sakthivel et al	
	Right side (median (IQR))	Left side (median (IQR))	Right side (median ± SD, range)	Left side (median ± SD, range)
Parotid SG	12.1 (10.2–14.6)	12 (10–15.1)	14.3 ± 8.3 (4.4–40.3)	13 ± 8.5 (4.3–45.7)
Submandibular SG	12.4 (10.2–15)	12.1 (10.4–15.4)	15.2 ± 8.5 (5.1–37)	15.5 ± 8.5 (6.1–38.1)
Sublingualis SG	4.3 (2.8–6.2)	4.4 (2.9–6.2)	8.6 ± 6.4 (2.1–34.3)	9.6 ± 6.4 (2.5–32.8)
Tubarial SG	4.1 (3.4–4.8)	4.3 (3.2–4.9)	6.6 ± 3.3 (2.5–13.5)	7.9 ± 2.7 (1.2–11.7)

*IQR* interquartile range, *SG* salivary glands, *SUVmax* maximum standardized uptake value, *SD* standard deviation

Still, the relevance of the TSGs regarding xerostomia remains debatable. The normal saliva production is around 0.6 L per day, of which approximately 90% of the fluid secretion is produced by the major salivary glands and only 10% by the minor salivary glands [19]. However, a relative large fraction of the salivary mucins, which provides lubrication to the oral surfaces, is produced by the minor salivary glands [19]. Xerostomia occurs when the unstimulated saliva secretion decreases by 40–50% of the baseline value, which corresponds to the dysfunction of at least one major salivary gland [19]. Yet, in some cases, xerostomia still occurs without salivary gland hypofunction, which might suggest that xerostomia may be caused by changes in saliva composition [19]. However, it is still unclear which saliva component(s) contributes to xerostomia. Furthermore, the severity of xerostomia might not be proportional to the PSMA uptake in the different salivary glands because xerostomia could be caused by the lack of multiple saliva components produced by the different salivary glands.

The TSGs were first described by Valstar et al. [13] as a “new” organ-at-risk with regard to high-dose EBRT. As the TSGs are located in the posterior nasopharynx, they will most likely be in the field of radiation during EBRT which ultimately will lead to toxicity. Therefore, the TSGs were considered clinically relevant, especially in the field of oncology. The same concern for toxicity applies for PSMA RNT due to the PSMA expression in the TSGs. After the radiolabeled PSMA-ligand is bound to the PSMA expressing cells, the radionuclide deposits its specific radiation (either alpha or beta particles) and causes DNA single- or double-stand breaks which leads to cell apoptosis [20]. Thus, PSMA RNT targets the lesions at a cellular level rather than at an anatomical level compared to EBRT [21]. Several studies have already evaluated different ways to protect the salivary glands (e.g., ice packs, dilatation, saline irrigation, prednisolone or folic glutamate tablets) during RNT which suggested possibilities to reduce salivary glands toxicity and to achieve significant improvement in quality of life [22, 23]. Interestingly, a mice study [24] showed that the uptake of ^177^Lu-PSMA-617 in the salivary gland and kidneys can be reduced substantially while maintaining enough uptake in the tumor by adding cold PSMA-11. As promising as PSMA RNT is, further research is still needed to limit salivary glands toxicity.

Although our study was not the first to quantitatively assess the TSGs on ^68^Ga-PSMA-11 images, our study provides additional necessary insights which are unmet in current literature. First, our patient population was larger compared to the patient population analyzed by Sakthivel et al. [15]. Second, Sakthivel et al. [15] only imaged the head-and-neck area and, therefore, needed to consider the left C1 and C2 paraspinal muscle as standard background. While our scan protocol covered the whole-body and could consider the thoracic aorta blood pool as standard background which might provide a more accurate correction of the SUVmax. Furthermore, patients by Sakthivel et al. [15] received a reduced amount of ^68^Ga-PSMA-11 activity to limit radiation exposure, while patients in the current study received the standard amount of ^68^Ga-PSMA-11 activity which provides a better representation of the actual distribution of PSMA uptake in the whole body. Lastly, by adding the TO-PSMA analysis, the gland function of the salivary glands could be estimated more precisely, and therefore, a more robust comparison could be made.

This study has several limitations. First, similarity between the SLSGs and the TSGs were solely based on the measurements derived from the ^68^Ga-PSMA-11 PET/CT images. With the addition of histological data, a more robust comparison could have been made between the SLSGs and the TSGs. However, to biopsy every salivary gland in large numbers seems not feasible. As histological data were not available in our database, histological comparison was not further explored in this study. Second, the measured PSMA uptake may be disproportional towards the saliva production which was not taken into account in this study. However, we aimed to approximate the salivary function by adding the TO-PSMA analysis. Still, the results should be interpreted with caution. Lastly, the ^68^Ga-PSMA-11 PET/CT data were analyzed in retrospect which could have led to selection bias.

In this study, first steps have been taken toward better understanding of the quantitative contribution of the TSGs towards the total salivary gland function. PSMA RNT and EBRT have been increasingly applied for the treatment of patients with cancer. These treatments usually result in xerostomia as it is one of the most common observed adverse events of EBRT in patients with head-and-neck cancer or brain metastasis, and PSMA RNT in patients with prostate cancer. Although most of the patients only experience mild symptoms such as a dry oral sensation or thickening of the saliva, some patients need to alter their dietary intake. In the worst case, even tube feeding is indicated. Despite the differences in the degree of burden between patients, xerostomia ultimately impacts the quality of life negatively. As the TSGs only contribute to 4.3% of the total salivary glands function, future research in general should be focused on minimizing toxicity in the major salivary glands. Several preclinical studies have shown promising newly developed PSMA-targeting ligands which lead to reduced PSMA uptake in the salivary glands [25–27]. In the field of radiotherapy, studies on toxicity reduction were mainly focused on dose constraints to the healthy tissues surrounding the target using different delineation techniques [28–30]. Ultimately, first steps have been taken towards treatment optimization; however, the challenge lies in the translation from preclinical research to the clinic application.

## Conclusion

In conclusion, the TSGs were visible on 95% of the ^68^Ga-PSMA PET/CT scans. Quantitatively, the SUVmean, SUVmax and TO-PSMA of the TSGs were most comparable to the SLSGs and contributed only 4.3% to the total salivary glands function. However, the measured PSMA uptake may be disproportional towards the saliva production. Therefore, future studies should focus on the relation between PSMA uptake and salivary function before and after PSMA therapy.

## Data Availability

The datasets generated during and/or analyzed during the current study are available from the corresponding author on reasonable request.
